# Extratemporal Facial Nerve Interconnections and Trunk’s Variability: A Systematic Review with Meta-Analysis

**DOI:** 10.3390/diagnostics14171862

**Published:** 2024-08-26

**Authors:** George Triantafyllou, Christos Tsiouris, Ioannis Chrysanthou, Ioannis Paschopoulos, George Tsakotos, Theodosis Kalamatianos, Maria Piagkou

**Affiliations:** 1 Department of Anatomy, School of Medicine, Faculty of Health Sciences, National and Kapodistrian University of Athens, 11527 Athens, Greece; tb.christos@gmail.com (C.T.); ioannischryssanthou@gmail.com (I.C.); johnpascho@gmail.com (I.P.); gtsakotos@gmail.com (G.T.); 2 Department of Neurosurgery, Evangelismos Hospital, School of Medicine, Faculty of Health Sciences, National and Kapodistrian University of Athens, 10676 Athens, Greece; tkalamatian@med.uoa.gr

**Keywords:** facial nerve, variation, branching pattern, interconnections, evidence-based anatomy

## Abstract

Background: The present systematic review with meta-analysis is a significant contribution to the understanding of the morphological variability of the facial nerve (FN) extratemporal segment, i.e., the facial trunk (FT) variability, its division, and terminal branching patterns. The study also provides a comprehensive overview of the clinical significance of the FN extracranial division. Methods: Four online databases were utilized to conduct the systematic review according to evidence-based anatomy guidelines. A meta-analysis of the studies included was carried out using R programming software. The combined prevalence of the FN variants was calculated, along with subgroup and cumulative analysis. Results: From the systematic review, 29 studies were retrieved as eligible for our initial purpose. However, 19 studies followed the same classification system and were selected for the meta-analysis, with a total sample of 2453 nerves. The most common pattern of the FN morphology was the FT bifurcation (typical pattern), with a pooled prevalence of 94.1% and a single interconnection (IC) between the temporofacial and cervicofacial branches (23.1% pooled prevalence). Two ICs between these branches were the rarest pattern (8.9% pooled prevalence). Conclusions: Our findings underscore the extensive morphological variability of the FN extratemporal anatomy, which has led to confusion among researchers. While several classification systems have been developed, none accurately represent the typical and variant anatomy. Our meta-analysis provided a small range between 8.9–23.1% for the rarest and most common pattern; thus, diversity is the rule. Therefore, it is not safe to conclude the typical morphology of FN extratemporal anatomy for its whole distribution before the FT’s division (proximally) and its terminal branches (distally). Nevertheless, the bifurcation of the FT can be considered the typical morphology, and it is far more constant than the distal branching pattern. These findings have significant implications for surgical procedures, particularly parotidectomy, where surgeons must exercise utmost caution due to the potential clinical implications of FN injury.

## 1. Introduction

The facial nerve (FN) is clinically essential and well-documented. It exits in the skull through the stylomastoid foramen and gives off preparotid branches, including the posterior auricular nerve, branches to the posterior belly of the digastric, and the stylohyoid muscle. The FN then continues as a trunk, the so-called facial trunk (FT), and further divides within the parotid gland into temporofacial and cervicofacial segments, which supply the facial expression muscles through their terminal branching pattern. The terminal branches are temporal or temporofrontal, the zygomatic, the buccal, the mandibular, and the cervical, essential for facial functionality [1]. However, Bergman et al. highlighted the challenge of defining the FT division due to possible variants like bifurcations, trifurcations, or multiplications, which add complexity to the FN pattern [1]. Rana et al. [2] reported that the FT division may appear as a single trunk, which is rare, only in 2% of the cases.

The FN is arranged in a complex network with many ending branches [3,4] and multiple interconnections (ICs) between the FN upper division and the “buccal component”, as expressed by Hovelacque [4] and McCormack et al. [5]. The ICs are notably prevalent within the temporofacial division branches. This prevalence is attributed to the division’s extensive branching and plexiform nature. Conversely, with its limited branch supply, the FN cervicofacial division is associated with a lower frequency of ICs [6]. Several anatomists have attempted to create a classification system to simplify and organize the FN branching patterns observed during dissection [5,7]. The extracranial branching pattern of the FN has been studied using various classification systems based on the type and number of ICs. Pascual et al. [6] developed a twelve-type classification system based on the analyses of 38 FNs, which aimed to unify different proposed classifications [8,9,10]. However, due to the complexity of the FN extracranial segment distribution, there may be variants in branching patterns that make systematic classification challenging.

The classification system proposed by Davis et al. [8] (six morphological types) has been widely used, while different FN classifications include six [7] to eight morphological types [5]. Davis et al. [8], after investigating 350 FNs, proposed a classification based on the presence of ICs between the FN terminal branches. Subsequently, in 1987, Katz and Catalano [9] described a five-typed classification considering the origin and the number of buccal branches (Table 1). Following these two widely recognized classifications, Kopuz et al. [10] developed a classification based on double FT types, and Kwak et al. [11] developed another based on the buccal branch origin. All these efforts to classify FN branching patterns reflect the significant heterogeneity when facing possible FN variants.

The FN branching pattern is determined during the first three months of prenatal development and continues to develop until approximately four years after birth. The proximal extratemporal branches (temporofacial and cervicofacial divisions) are formed at the end of the 7th week, followed by the formation of the distal branches (five major peripheral subdivisions) at the end of the 8th week. The FN terminal branches start to appear during the early part of the 8th week and are well established by the end of that week. By 12 weeks, the branching pattern becomes very complex, and parotid ductules grow between many FN branches, connecting the superficial and deep portions of the gland. Additionally, at this stage, the facial muscles reach their definitive position.

The complex pattern of the extracranial FN branching can pose challenges in parotid gland surgery, potentially hindering the removal of lesions and increasing the risk of nerve injury [12]. Damage to any of these branches during parotid surgery may result in facial paralysis [13]. Additionally, variants in FN anatomy may increase the likelihood of post-surgical facial paralysis if the surgeon is not familiar with these variants [14].

This evidence-based systematic review with meta-analysis explores potential variations in the terminal branching pattern, primary division branches, and FT.

## 2. Materials and Methods

### 2.1. Literature Search Analysis

The systematic review, with meta-analysis, adhered to the guidelines set forth by the Evidence-based Anatomy Workgroup [15] and the PRISMA 2020 Guidelines [16]. Four independent reviewers conducted the literature search and data extraction. The results were then compared, and the co-authors resolved any discrepancies. The search terms “facial nerve”, “branching pattern”, “branches”, “course”, “variation”, “origin”, “anatomical study”, and “surgical study” were used in various combinations across PubMed, Google Scholar, Scopus, and Web of Science databases up to May 2024. An example of the search keywords used is outlined in Table 1.

Studies that included FN extracranial branching pattern data were selected for the current evidence-based systematic review. There were no language or date restrictions, but case reports, animal studies, conference abstracts, and letters to the editor were excluded. Studies with irrelevant, insufficient, or incomplete data were only included if they met the inclusion criteria. Additionally, other sources were searched for eligible articles, starting with an investigation of the grey literature and followed by a hand search of significant anatomical journals (Annals of Anatomy, Journal of Anatomy, Anatomical Record, Clinical Anatomy, Surgical and Radiological Anatomy, Anatomical Science International, Folia Morphologica, and Anatomy Cell Biology). Lastly, the references of all included studies were reviewed for additional articles. Microsoft Excel was used to evaluate the extracted data. The extracted data were the following: date of publication, country, type of study (cadaveric or surgical), total sample, gender (male or female), side (left or right), FT division pattern (bifurcation, trifurcation or multiplication), and FN terminal branching pattern ICs. For publication bias, according to the AQUA Tool, five domains with questions and possible answers of “Yes, No, or Unclear” provide the potential risk of bias as “Low, High, or Unclear” [17].

### 2.2. Facial Nerve (FN) Classification

Upon conducting a detailed literature search, most studies used Davis et al.‘s [8] classification of the FN branches. This classification system included Types I to VI, each describing the different patterns of ICs between the FN branches. Type I had no ICs between terminal branches, type II had several ICs between the temporofacial branches, type III had a single IC between the temporofacial and the cervicofacial division branches, type IV was a combination of Types II and III, type V had two ICs between the temporofacial and cervicofacial division branches, and type VI had multiple ICs between the FN terminal branches. The statistical analysis only included these studies because it was not possible to combine results from different classification systems. The classification by Davis et al. [8] was chosen because most of the eligible studies (19 out of 29) reported their results using this classification. Some studies used Katz and Catalone’s [9] classification, and others used their classification systems. We will further discuss all these approaches.

### 2.3. Meta-Analysis Process

The statistical analysis utilized the open-source R programming language (version 4.3.3) and the RStudio software (version 2023.12.1+402). The following packages were used for the analysis: “meta”, “metafor”, and “dmetar” [18,19,20,21,22]. Prevalence meta-analysis was undertaken based on the inverse variance method and the random effects model using the Freeman–Tukey double arcsine transformation, the DerSimonian–Laird estimator for the between-study variance tau^2^, and the Jackson method for the confidence intervals of tau^2^ and tau. The presence of heterogeneity across the included studies was evaluated using Cochran’s Q statistic (*p*-value). Based on the Higgins I^2^ statistic, the heterogeneity was quantified as minor (0% ≤ I^2^ < 25%), low (25% ≤ I^2^ < 50%), moderate (50% ≤ I^2^ < 75%), and high (I^2^ ≥ 75%). Subgroup analyses were performed to test the effect of the subjects’ geographical region (continent of origin) and the study’s design (cadaveric or surgical) on the estimated prevalence.

To examine the presence of the small-study effect [23] a cumulative meta-analysis [21] sorting studies by sample size from highest to lowest and a regression test for funnel plot asymmetry (mixed-effects meta-regression model, predictor: sample size) were conducted. The sample size was used to measure precision on the *y*-axis of the funnel plot, as suggested by Hunter et al. [20] for the meta-analysis of proportions.

Outlier analyses were conducted to detect possible outlier studies with outlying prevalences that distort the estimated pooled prevalences. The pooled estimates were recalculated after excluding the outliers. Influence analyses were applied to identify possible influential studies and determine whether an outlier study is also an influential study with a large impact on the estimated prevalence [22,23,24]. Statistical significance was denoted by a *p*-value less than 0.05. Moreover, Fu et al. [25] proposed that four studies per variable should be achieved for subgroup analysis.

## 3. Results

### 3.1. Study Selection

**Figure 1:**
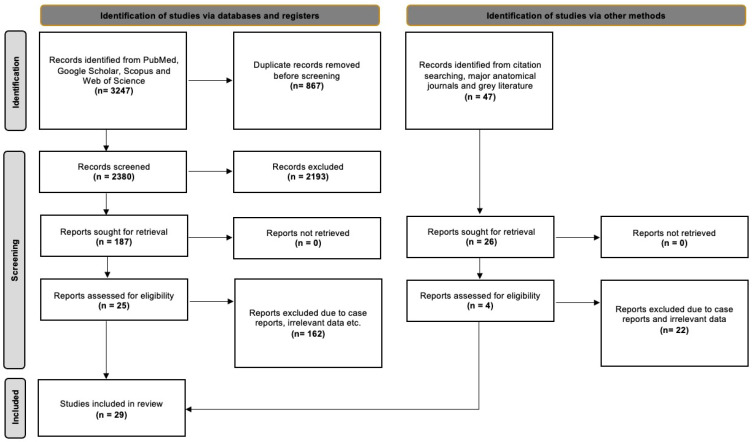
PRISMA 2020 flow diagram [16].

The database searches yielded 3247 results, which were then exported to Mendeley version 2.10.9 (Elsevier, London, UK). We first checked for duplicate entries and then reviewed the titles and abstracts. After excluding irrelevant papers based on the title and abstract, we proceeded to screen the full text of 187 studies. Of these, 25 studies were suitable for addressing our systematic review questions. Additionally, we identified 47 potentially eligible studies from references, the grey literature, and significant anatomical journals, out of which four studies met all the criteria. In total, 29 studies were included in the current evidence-based systematic review. Nineteen of them were classified according to Davis et al. [8] and were included in the statistical meta-analysis. The detailed selection process is outlined in Figure 1, following the PRISMA 2020 flow diagram.

### 3.2. Study Characteristics

The review included 29 studies, encompassing 2453 nerves. Nineteen were based on anatomical dissections, while the remaining 10 focused on intraoperative findings. On average, each article examined 84.58 nerves. Regarding geographic distribution, 17 articles were related to the Asian population, six to the European population, five to the American population, and one study focused on the African population (see Table 2).

### 3.3. Facial Nerve (FN) Trunk Morphology and Branching Pattern Interconnections (ICs) Pooled Prevalence

**Figure 2:**
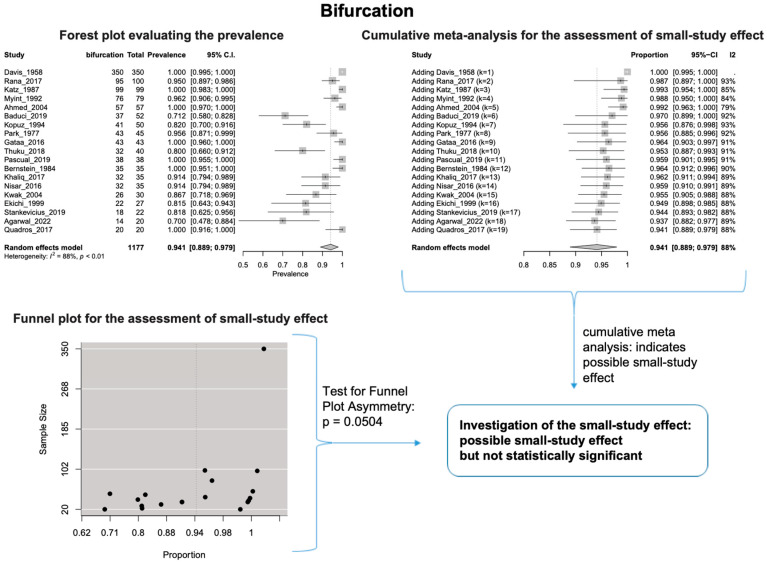
Bifurcation: Forest plot evaluating the prevalence, cumulative meta-analysis assessing the small-study effect, and funnel plot assessing the small-study effect [2,6,8,9,10,11,26,27,28,29,30,31,32,33,34,35,36,37,38,39,40,41,42,43,44,45,46,47].

**Figure 3:**
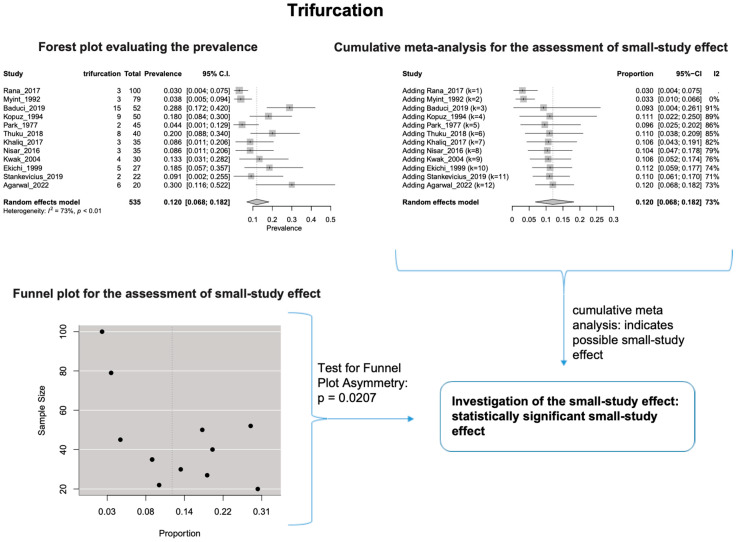
Trifurcation: Forest plot evaluating the prevalence, Cumulative meta-analysis assessing the small-study effect, and funnel plot assessing the small-study effect [2,6,8,9,10,11,26,27,28,29,30,31,32,33,34,35,36,37,38,39,40,41,42,43,44,45,46,47].

The estimated heterogeneity was statistically significant (*p*-value < 0.01). The FNT bifurcated in 94.1% [95% CI: 88.9–97.9%; Heterogeneity: *p*-value < 0.01 and I^2^ = 88.0% (high degree)] (Figure 2) and trifurcated in 12.0% [95% CI: 6.8–18.2%; Heterogeneity: *p*-value < 0.01 and I^2^ = 72.7% (moderate degree)] of cases (Figure 3).

Based on the cumulative meta-analysis, as small sample studies are added, the estimated cumulative prevalence of the bifurcated FN tends to decrease (Figure 2). In contrast, the estimated prevalence of the trifurcated FN tends to increase (Figure 3). Therefore, the cumulative meta-analysis indicates the presence of small-study effect on the estimated prevalence for both bifurcated and trifurcated FN morphology. Using the sample size as the measure of precision [20], it is considered that larger sample sizes provide more precise estimates of the prevalence. Therefore, the cumulative meta-analysis results indicate that true prevalence in the population is possibly higher than the estimated 94.1% for bifurcated FN and lower than the estimated 12.0% for trifurcated FN. Based on the regression tests, asymmetry in the funnel plots was estimated as statistically significant only for the trifurcated FN (*p*-value = 0.0207 < 0.05), showing the considerable impact of small studies on the estimated prevalence of trifurcated FN. However, the *p*-value of the test for funnel plot asymmetry for the bifurcated FN was estimated as 0.0504, which is almost equal to the statistically significant level of 0.05. The results indicate that small sample size studies have affected the estimated prevalence for both bifurcated and trifurcated FN morphology. Therefore, further studies with larger sample sizes are required to estimate the true prevalence accurately.

**Figure 4:**
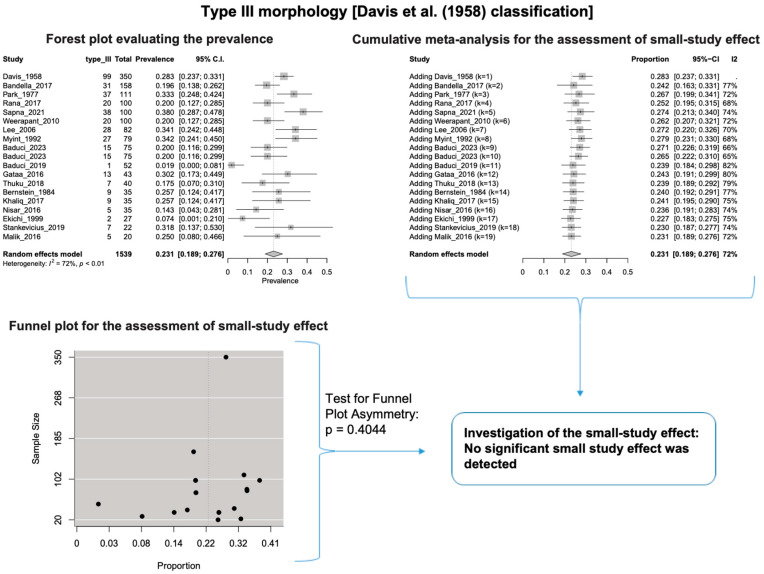
Type III morphology: Forest plot evaluating the prevalence, cumulative meta-analysis for assessing the small-study effect, funnel plot for assessing the small-study effect [2,6,8,9,10,11,26,27,28,29,30,31,32,33,34,35,36,37,38,39,40,41,42,43,44,45,46,47].

**Figure 5:**
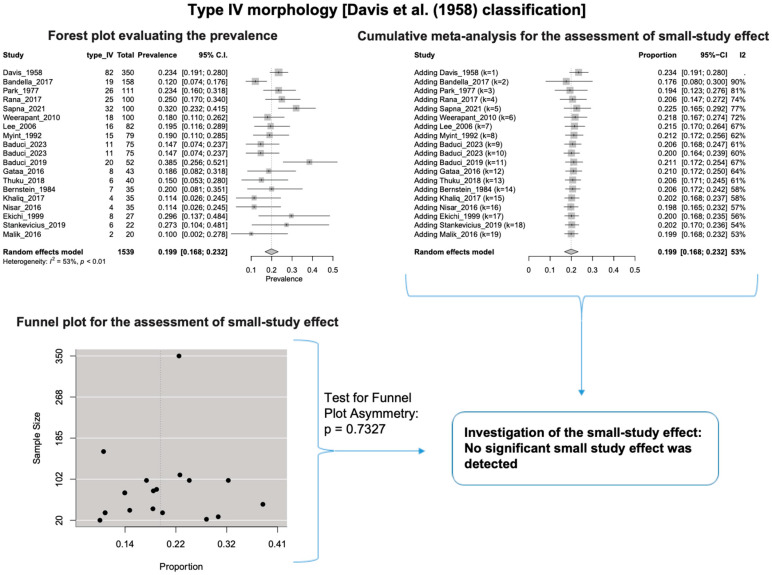
Type IV morphology: Forest plot evaluating the prevalence, cumulative meta-analysis for assessing the small-study effect, funnel plot for assessing the small-study effect [2,6,8,9,10,11,26,27,28,29,30,31,32,33,34,35,36,37,38,39,40,41,42,43,44,45,46,47].

**Figure 6:**
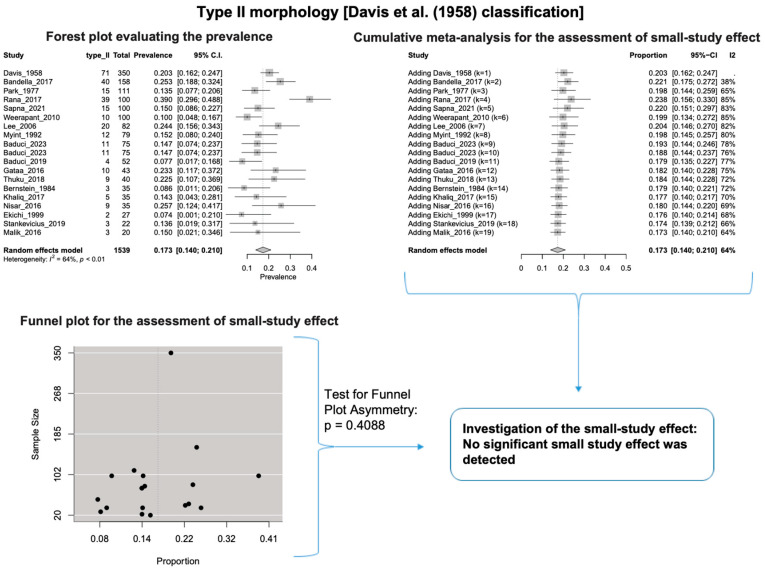
Type II morphology: Forest plot evaluating the prevalence, cumulative meta-analysis for assessing the small-study effect, funnel plot for assessing the small-study effect [2,6,8,9,10,11,26,27,28,29,30,31,32,33,34,35,36,37,38,39,40,41,42,43,44,45,46,47].

**Figure 7:**
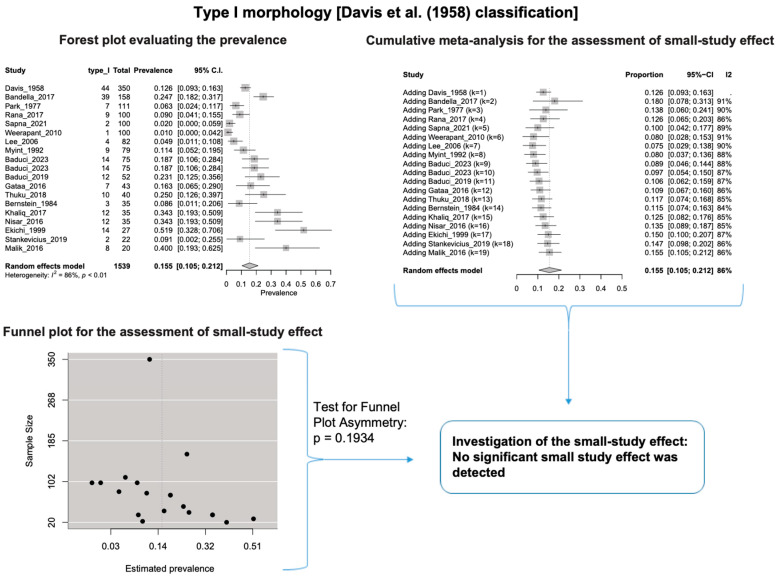
Type I morphology: Forest plot evaluating the prevalence, cumulative meta-analysis for assessing the small-study effect, funnel plot for assessing the small-study effect [2,6,8,9,10,11,26,27,28,29,30,31,32,33,34,35,36,37,38,39,40,41,42,43,44,45,46,47].

**Figure 8:**
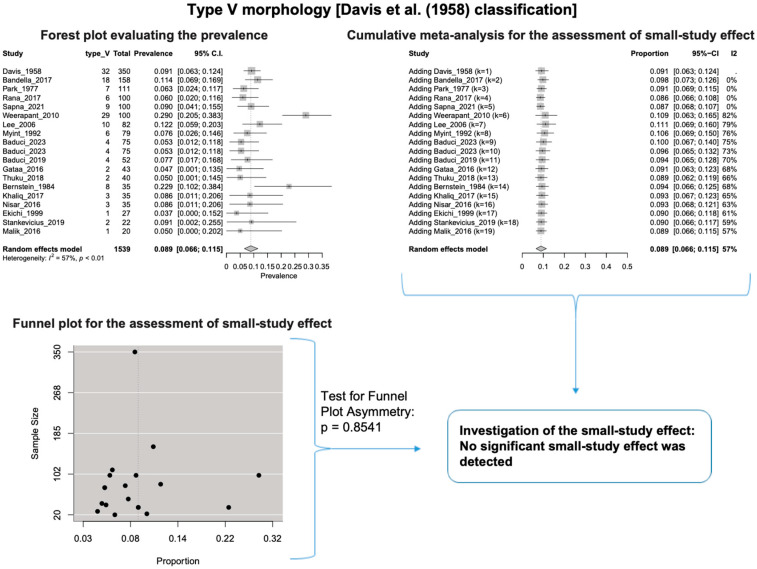
Type V morphology: Forest plot evaluating the prevalence, cumulative meta-analysis for assessing the small-study effect, funnel plot for assessing the small-study effect [2,6,8,9,10,11,26,27,28,29,30,31,32,33,34,35,36,37,38,39,40,41,42,43,44,45,46,47].

**Figure 9:**
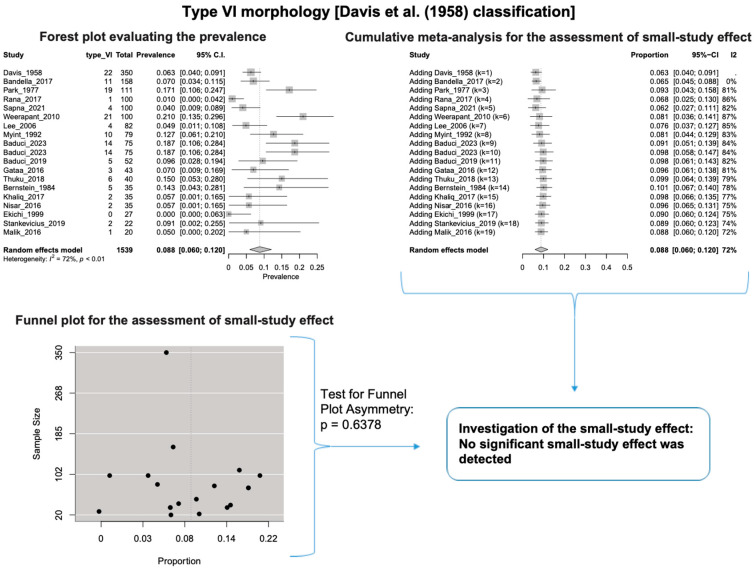
Type VI morphology: Forest plot evaluating the prevalence, cumulative meta-analysis for the assessment of the small-study effect, funnel plot for the evaluation of the small-study effect [2,6,8,9,10,11,26,27,28,29,30,31,32,33,34,35,36,37,38,39,40,41,42,43,44,45,46,47].

A single IC between the temporofacial and cervicofacial division branches (type III, according to Davis et al. [8]) had a pooled prevalence of 23.1% [95%CI: 18.9–27.6%; Heterogeneity: *p*-value < 0.01 and I^2^ = 72.1% (moderate degree)] (Figure 4). A combination of several ICs between the temporofacial branches and the temporofacial and cervicofacial division branches (type IV, according to Davis et al.) had a pooled prevalence of 19.9% [95% CI: 16.8–23.2%; Heterogeneity: *p*-value < 0.01 and I^2^ = 52.7% (moderate degree)] (Figure 5). Several ICs between the temporofacial branches (type II) had a pooled prevalence of 17.3% [95%CI: 14.0–21.0%; Heterogeneity: *p*-value < 0.01 and I^2^ = 64.3% (moderate degree)] (Figure 6). No ICs between terminal branches (type I) had a pooled prevalence of 15.5% [95% CI: 10.5–21.2%; Heterogeneity: *p*-value < 0.01 and I^2^ = 86.4% (high degree)] (Figure 7). Two ICs between the temporofacial and cervicofacial division branches (type V morphology, according to Davis et al. [8]) had a pooled prevalence of 8.9% [95%CI: 6.6–11.5%; Heterogeneity: *p* value < 0.01 and I^2^ = 56.8% (moderate degree)] (Figure 8). Multiple ICs between the FN terminal branches (type VI morphology, according to Davis et al. [8]) had a pooled prevalence of 8.8% [95% CI: 6.0–12.0%; Heterogeneity: *p*-value < 0.01 and I^2^ = 72.4% (moderate degree)] (Figure 9). The cumulative meta- analyses and the regression tests for funnel plot asymmetry yielded no small-study effect.

Table 3 summarizes the subgroup analysis of each FN morphological type. A statistically significant difference was identified between nationalities (*p*-value = 0.0023) and studies’ type (*p*-value = 0.0017) for the non-ICs between the terminal branches’ morphology (Type I). However, the nationality subgroup analysis should be carefully considered because a minimum of four studies per subgroup was not achieved for each nationality, as Fu et al. [25] suggested for a (categorical) subgroup variable.

The results of the outlier and influence analyses are summarized in Table 4. Influential outlier studies, with substantial impact on both the estimated prevalence and heterogeneity, were detected in Davis et al. type II [2], type III [27], and type V [41] morphologies, and therefore the estimated prevalences of these morphologies may be distorted. The re-calculation of the prevalence after excluding the outliers for the bifurcated FN morphology (four outlier studies: Davis et al. [8], Katz et al. [9], Baduci et al. [27], and Agarwal et al. [46]) yielded a slight increase in the estimated mean prevalence [(0.9447 − 0.9412)/0.9412 ≈ +0.4% change], which is in line with the cumulative meta-analysis results that true prevalence in the population is possibly higher than the estimated 94.1% for bifurcated FN. However, after excluding one outlier study [2] that was detected in trifurcated FN morphology, the estimated mean prevalence increased by (0.1340 − 0.1201)/0.1201 ≈ 11.6%, which contrasts with the cumulative meta-analysis results that the actual prevalence of trifurcated FN in the population is possibly lower than the estimated 12.0%. The outlier study, Rana et al. [2], has the largest sample size among the included studies for the trifurcated FN morphology. Thus, the exclusion of this study increases the impact of smaller studies on the overall estimate, leading to an increase in the pooled prevalence. Therefore, further larger-scale studies are required for a more precise estimation of the true prevalence. The influence analysis plots are presented in the Supplementary Materials. Influential studies are red-colored in the influence diagnostics plots (Supplementary Figures S1–S8).

## 4. Discussion

### 4.1. Morphological Variability of Facial Nerve Branching Pattern Interconnections

The most commonly used classification for studies is the one developed by Davis et al. [8] in 1958. This classification is based on the ICs between the FN terminal branches. However, it does not fully consider variants in the origins and number of these branches. Despite its limitations, this classification was used for the current meta-analysis due to its widespread use in previous studies. As a result of the analysis, the prevalence of each morphological type was found to be in the following order: III, IV, II, I, V, and VI. The prevalences of each type are very similar, indicating a wide range of morphological variants in the FN terminal branching pattern, making it difficult to establish a typical FN branching pattern.

In our meta-analysis, we calculated the combined prevalence of each type based on the Davis et al. [8] classification system. We conducted subgroup analyses by nationality and study type (see Table 3). It is important to note that small study sizes, outlier studies, or significant diversity in the data influenced some of our findings. For instance, for Davis et al.’s [8] Type II, III, and V, one particular study stood out and had a notable impact on the calculated prevalence of each type. Statistical analysis is standard in anatomical systematic reviews and meta-analyses [15]. To address potential confusion and errors, we re-evaluated the combined prevalence of these types using the leave-one-out method, and the results are summarized in Table 4.

In 1987, Katz and Catalano modified the classification of Davis et al. [8], but only five studies used their classification. They based their classification on the FN terminal branches, the ICs, the buccal branch origin, and the FTs within the parotid gland. According to Katz and Catalano [9], Type I (24%) corresponded to a single IC in either the zygomatic or marginal mandibular branch. Type II (14%) presented with an IC loop between the zygomatic and buccal branches, similar to Davis et al. [8] Type II. Type III (44%) was the most common and had a major IC between the buccal branch and the zygomatic or mandibular nerves. Type IV (14%) corresponded to the “multiple loop pattern” with multiple IC loops between the zygomatic, buccal, and marginal mandibular branches. Type V (3%) was the rarest, with two FTs (major and minor) within the parotid gland. Each type had several subtypes in the Katz and Catalano [9] classification, highlighting the vast morphological variability. Although this detailed classification seems more comprehensive than that of Davis et al. [8], it was not possible to use it for the current meta-analysis due to the limited number of studies that adhered to the Katz and Catalano [9] classification.

According to Bergman’s Comprehensive Encyclopedia of Human Anatomic Variation [1], a few parameters might affect the significant differences between the FN branching patterns. Firstly, the dissection method and the essential equipment are essential, as smaller ICs could be destroyed during dissection. Secondly, there are possible differences between nationalities, while the current meta-analysis did not retrieve statistically significant associations except for Type I morphology. Thirdly, the smaller samples could affect the results, though the current meta-analysis did not find a small-study effect except for Type VI morphology. Lastly, the terminology and correct identification of branches may cause differences between the studies [1].

The classic anatomy textbooks describe the FN’s five branches, corresponding to Davis et al.‘s Type I [8]. This means that the FN’s branching pattern exhibits considerable variability in its morphology. Pascual et al. [6] conducted a dissection and categorized 38 FNs to better understand the complex anatomy of FN branches. They classified the branching pattern into a comprehensive system of 12 types, incorporating the classification systems of Davis et al. [8], Katz and Catalano [9], and Kopuz et al. [10]. They presented their findings, highlighting the FT, terminal branches, patterns, and ICs.

### 4.2. Morphological Variability of Facial Nerve Trunk (FNT) and Branches

Sometimes, a trifurcation or even a multifurcation of the FT is documented [48,49,50]. The reported incidence of FT bifurcation is 80%, the trifurcation is 14%, and other variants are found in 6% of cases [48,49,50]. Babuci et al. [26] recorded an FT bifurcation in 84% of cases, trifurcation in 6.6%, and more complex divisions in 9.4%. In cases of a dehiscent facial canal, a double or triple FT is present [48,49,50].

In a rare dissection report by Kilic et al. [49], they observed a double FT emerging from the stylomastoid foramen and the petrotympanic fissure. Two buccal branches of the FN accompanied this variant. In another case, Reija et al. [50] identified an FT duplication during a superficial parotidectomy to remove a pleomorphic adenoma. After exiting through the stylomastoid foramen, the FT split into two main divisions before merging back together just before entering the parotid gland. It is essential to be cautious during procedures that involve FT manipulation and isolation, as damage to it can lead to significant nerve injury, and care should be taken during dissection to avoid this. Zhou et al. [51] also reported finding an FT fenestration by the stylomastoid artery in two patients.

Poutoglidis et al. [52] observed that the FN zygomatic branch was absent. They also noted that the FT temporofacial division consists of temporal and buccal branches, which coexist with a plexus formation between the anterior temporal and posterior buccal branches. In their study on the marginal mandibular branch of the FN, Balagopal et al. [53] found that 79.7% of the patients had a single branch, 12.9% had two branches, 6.9% had three branches, and one patient had four branches. Additionally, they observed ICs between the marginal mandibular branch of the FN and the FN cervical branch in 49 patients. Babuci et al. [27] observed variants in the number of cervical branches of the FN, ranging from one to five. They found the following distributions: 61.3% had one branch, 28% had two branches, 6.7% had three branches, 2.7% had four, and 1.3% had five branches. In the study by Tsai et al. [54] on 35 cadaveric hemifaces, they recorded that 37.15% had two buccal branches, 48.59% had three branches, and 5.7% had four buccal branches. Additionally, they noted no ICs between the upper and lower buccal branches in 31% of the hemifaces.

### 4.3. Facial Nerve Interconnections with Other Nerves and Relationship with the Retromandibular Vein

In addition to studying the FN branching pattern, researchers also examined the relationship between the retromandibular vein (RMV) and the ICs with other nerves. Some studies, such as Laing and McKerrow [43] and Touré and Vacher [55], have looked into the relationship between the FN and the RMV. Piagkou et al. [56] proposed a classification system for the FN-RMV relationship, which included Type I (typical RMV deep position to the FN), Type II (RMV superficial position to the FN), and Type III and IV (RMV variants such as fenestration and duplication, with the FN in various relationships according to the RMV variants). Unfortunately, there were not enough studies on this relationship to be included in the current meta-analysis. Kininy et al. [57] discovered an unusual variant of a superficial temporal vein lying superficial to the FN, occurring within the parotid gland. These findings hold importance for surgical approaches to the mandible for condylar trauma or osteotomy surgery.

Diamond et al. [58], Shoja et al. [59], and Tubbs et al. [1] have reported various ICs involving the FN with other nerves. Diamond et al. [58] and Shoja et al. [59] identified thirty-two ICs involving the FN, such as ICs with the superior or inferior vestibular nerve, glossopharyngeal nerve, auricular branch of the vagus nerve, auriculotemporal nerve, mental nerve, and great auricular nerve. Tubbs et al. [1] also discussed the IC of the FN with various nerves. Gulati et al. [60] also discovered an interesting IC between the FN and the ansa cervicalis. Their research revealed that the FN cervicofacial division extended distally to form an IC with the distal loop of the ansa cervicalis. This unexpected connection has implications for potential facial paralysis if either nerve is injured, highlighting the need for meticulous care during neck surgeries.

### 4.4. Clinical Significance of the Facial Nerve (FN)

The FN has been extensively studied due to its high clinical significance. Knowledge of the morphological (FT and branching pattern) and topographical anatomy (relationships with the adjacent structures) is paramount for facial interventions, especially for parotid surgery [61]. The successful identification, dissection, and preservation of FN is considered adequate during parotid gland tumor removal and surrounding salivary tissue [9]. Moreover, salivary gland surgery, head and neck traumas, and aesthetic surgery correspond to operations that expose the FN to irreversible lesions [26]. Iatrogenic lesions to the FT or its branches could lead to either temporary or permanent palsy [61]. Katz and Catalano [9] predicted the result of possible lesions between its classification system. According to their classification, the Type I pattern is clinically significant due to the potential damage of any branch that will lead to paralysis of the supplied muscles. The Type III branching pattern, most commonly observed in their study, presents excellent safety to surgeons for dissection. Lastly, while Type V was the rarest one, it could lead to significant lesions because the surgeon should remember that, after the FΤ identification, another minor trunk could also exist [9]. Nowadays, intraoperative FN monitoring is an essential tool that contributes to safe surgical procedures, mainly when close dissection of the nerve is performed, such as skull base and middle ear or mastoid surgery [62]. Therefore, meticulous intraoperative dissection with monitoring offers the best chance to minimize the risk of injury. However, specific conditions require FN sacrifice for better oncological outcomes during tumor resection; in those cases, FN repair with grafts can repair the nerve’s functionality [63]. Adequate knowledge of FN landmarks is paramount during parotidectomy because FN preservation should be the surgeon’s goal [64]. Except for intraoperative lesions, FN may be affected by neurological lesions at different levels of its pathways [65]. Takezawa et al. [65] highlighted five clinical problems. Strokes or transient ischemic attacks, lesions at the fourth ventricle floor, acoustic neuroma, Bell’s palsy, and anesthesia within the parotid gland could affect the FN, leading to pathology [65].

During parotid surgery, the FN is at risk of injury, especially in large tumors where the nerve may be displaced [64]. Therefore, it is crucial to use nerve monitoring and mapping before making any incisions to prevent accidental damage [64]. Typically, intraoperative neuromonitoring is used during parotid surgeries to identify the FN branches [64]. The anterograde approach is a commonly used and safe method for dissecting the FN. This involves identifying the main trunk and carefully dissecting it, following its path and branches in an anterograde direction [64]. Several key landmarks, such as the stylomastoid foramen, the posterior digastric belly, the tympanomastoid suture, and the junction between the bony and cartilaginous ear canal, can help locate the main trunk of the FN [64]. However, it is important to note that the approach may vary depending on the tumor’s location [64]. By using the anterograde approach with neuromonitoring, surgeons can cautiously dissect the FN, considering its significant morphological variants, to avoid accidental intraoperative damage.

### 4.5. Limitations

Although the meta-analysis followed evidence-based procedures, it is essential to mention certain limitations. Significant heterogeneity was observed during subgroup analysis, a common challenge in anatomical meta-analyses. Nevertheless, the AQUA tool identified a high risk of bias in most of the included studies, as expected during an anatomical systematic review. This was highlighted during the development of the AQUA tool [17]. However, the biggest issue arose in the existing literature. As already highlighted, the FN extracranial anatomy has not been well studied, with few classification systems, which do not depict the significant morphological variability of the nerve. This fact was highlighted by the meta-analysis that described a range of 8.9–23.1% between the rarest and most expected variants; thus, conclusions about the FN typical and variant anatomy are unsafe. Moreover, influential outlier studies were detected in Davis et al. type II, type III, and type V morphologies [8], and therefore, the estimated prevalences of these morphologies may be distorted. Larger scale studies are required for more precise estimations, especially for bifurcated and trifurcated FN, as small sample size studies greatly impacted both estimated prevalences.

## 5. Conclusions

In the present systematic review with meta-analysis, the extracranial anatomy of FN was examined, focusing on FT variations and the existence of ICs between its terminal branches. The most common morphology was a single IC between the cervicofacial and temporofacial divisions, with a prevalence of 23.1%. The FT bifurcated and gave off terminal branches with a prevalence of 94.1%. Our review revealed significant confusion in the existing literature regarding FN extracranial anatomy due to the lack of standardized classification systems. The variability in FN terminal branches is extensive, emphasizing the need for surgeons to exercise caution when operating in this area. Nevertheless, the FT bifurcation is constant and can be considered the typical morphology.

## Figures and Tables

**Table 1:** Example of the keyword combinations used for the present systematic review.

Search Number	Search Term Combinations
1	((facial nerve) AND (branching pattern)) AND (variation)
2	(facial nerve) AND ((branches) OR (course) OR (origin)) AND (variation)
3	((facial nerve) AND (variation)) AND ((anatomical study) OR (surgical study))

**Table 2:** A summary of the characteristics of the eligible studies, including the risk of bias assessment based on the Anatomical Quality Assessment Tool (AQUA). NR—not reported.

Study	Population	Type of Study	Age Group	No. of Nerves	Risk of Bias
Babuci et al. [26]	Europe	Cadaveric	Adults	75	Low
Babuci et al. [27]	Europe	Cadaveric	Adults	52	High
Babuci et al. [28]	Europe	Cadaveric	Adults	75	High
Bandella et al. [29]	Europe	Cadaveric	NR	158	High
Bernstein and Nelson [30]	America	Cadaveric	Adults	35	High
Davis [8]	America	Cadaveric	NR	350	High
Ekichi [31]	NR	Cadaveric	Children	27	High
Gataa and Faris [32]	Asia	Surgical	NR	43	High
Khaliq et al. [33]	Asia	Surgical	Adults	35	High
Lee et al. [34]	Asia	Cadaveric	Fetuses and Stillborn infants	82	High
Malik [35]	Asia	Surgical	Adults	20	Low
Myint et al. [36]	Asia	Cadaveric	Adults	79	High
Park and Lee [37]	Asia	Cadaveric	Adults	111	High
Rana [2]	Asia	Cadaveric	Adults	100	High
Sapna [38]	Asia	Surgical and Cadaveric	Adults	100	High
Stankevicius and Sucholminov [39]	Europe	Cadaveric	Adults	22	Low
Thuku et al. [40]	Africa	Cadaveric	Adults	40	High
Weerapant et al. [41]	Asia	Cadaveric	Adults	100	High
Ahmed et al. [42]	Asia	Cadaveric	Adults	57	High
Alkan et al. [43]	Asia	Cadaveric	Adults	50	High
Babiker et al. [44]	America	Surgical and Cadaveric	NR	90	High
Katz and Catelano [9]	America	Surgical	NR	99	High
Kopuz et al. [10]	Asia	Surgical and Cadaveric	Children and Adults	50	Low
Kwak et al. [11]	Asia	Cadaveric	Adults	30	Low
Pascual et al. [6]	Europe	Cadaveric	Adults	38	High
Quadros et al. [45]	Asia	Cadaveric	NR	20	High
Agarwal et al. [46]	Asia	Surgical	NR	20	High
Alomar et al. [47]	Asia	Surgical	NR	460	High

**Table 3:** The results of the subgroup analyses on the effect of the subjects’ geographical region and the study’s design on the estimated prevalence. k, Number of studies combined; 95%-CI, 95% confidence interval; I^2^, Higgins I^2^ statistic. With bold letters appear the statistically significant results.

Morphology	Moderator: Categorical Predictor	Subgroups	k	Prevalence [95%-CI]	Heterogeneity: Quantification (I^2^)	*p*-Value of Test for Subgroup Differences
type I	Continent of origin	America	2	0.1181 [0.0867; 0.1533]	minor (0.0%)	**0.0023**
		Europe	5	0.2089 [0.1686; 0.2521]	minor (0.0%)	
		Asia	10	0.1200 [0.0574; 0.1995]	high (87.2%)	
		Africa	1	0.2500 [0.1263; 0.3973]	-	
	Study’s design	Cadaveric	13	0.1209 [0.0790; 0.1697]	high (81.5%)	**0.0017**
		Surgical	4	0.2965 [0.1904; 0.4141]	low (48.5%)	
type II	Continent of origin	America	2	0.1559 [0.0646; 0.2754]	moderate (66.4%)	0.7278
		Europe	5	0.1558 [0.0959; 0.2260]	moderate (62.1%)	
		Asia	10	0.1899 [0.1338; 0.2527]	moderate (73.4%)	
		Africa	1	0.2250 [0.1070; 0.3689]	-	
	Study’s design	Cadaveric	13	0.1756 [0.1336; 0.2217]	moderate (73.3%)	0.4803
		Surgical	4	0.2003 [0.1341; 0.2750]	minor (0.0%)	
type III	Continent of origin	America	2	0.2782 [0.2339; 0.3248]	minor (0.0%)	0.1019
		Europe	5	0.1660 [0.0864; 0.2636]	high (78.8%)	
		Asia	10	0.2783 [0.2284; 0.3310]	moderate (53.7%)	
		Africa	1	0.1750 [0.0705; 0.3103]	-	
	Study’s design	Cadaveric	13	0.2282 [0.1802; 0.2799]	moderate (74.9%)	0.7747
		Surgical	4	0.2370 [0.1663; 0.3153]	minor (0.0%)	
type IV	Continent of origin	America	2	0.2284 [0.1870; 0.2724]	minor (0.0%)	0.6255
		Europe	5	0.1953 [0.1121; 0.2938]	high (77.3%)	
		Asia	10	0.2027 [0.1654; 0.2426]	low (34.3%)	
		Africa	1	0.1500 [0.0534; 0.2798]	-	
	Study’s design	Cadaveric	13	0.1995 [0.1659; 0.2353]	moderate (52.0%)	0.0998
		Surgical	4	0.1330 [0.0776; 0.1990]	minor (0.0%)	
type V	Continent of origin	America	2	0.1406 [0.0329; 0.3007]	high (80.2%)	0.6532
		Europe	5	0.0794 [0.0529; 0.1101]	minor (0.0%)	
		Asia	10	0.0939 [0.0550; 0.1409]	moderate (70.4%)	
		Africa	1	0.0500 [0.0009; 0.1448]	-	
	Study’s design	Cadaveric	13	0.0969 [0.0665; 0.1318]	moderate (69.5%)	0.3996
		Surgical	4	0.0656 [0.0259; 0.1180]	minor (0.0%)	
type VI	Continent of origin	America	2	0.0849 [0.0221; 0.1785]	moderate (63.2%)	0.4733
		Europe	5	0.1218 [0.0692; 0.1855]	moderate (60.9%)	
		Asia	10	0.0773 [0.0375; 0.1280]	high (77.5%)	
		Africa	1	0.1500 [0.0534; 0.2798]	-	
	Study’s design	Cadaveric	13	0.1084 [0.0714; 0.1516]	high (78.1%)	0.1711
		Surgical	4	0.0591 [0.0214; 0.1098]	minor (0.0%)	

**Table 4:** The results of the outlier and influence analyses. k, Number of studies combined; Pr, Prevalence; 95%-CI, 95% confidence interval; I^2^, Higgins I^2^ statistic.

Morphology	Initial Estimation			Outlier Analysis	Influence Diagnostics	Re-Estimation with Outliers Removed			% Change	
	k	Pr [95%-CI]	Heterogeneity: Quantification (I^2^)	Outlier Studies “First Author_Year”	Influential Studies “First Author_Year”	k	Pr [95%-CI]	Heterogeneity: Quantification (I^2^)	Pr	I^2^
type I	19	0.1551 [0.1054; 0.2117]	high (86.4%)	“Sapna_2021” [38], “Weerapant_2010” [41], “Lee_2006” [34], “Ekichi_1999” [31]	-	15	0.1750 [0.1311; 0.2235]	moderate (73.6%)	+12.8%	−14.8%
type II	19	0.1733 [0.1395; 0.2098]	moderate (64.3%)	“Rana_2017” [2]	“Rana_2017” [2]	18	0.1631 [0.1363; 0.1916]	low (40.3%)	−5.9%	−37.3%
type III	19	0.2309 [0.1886; 0.2760]	moderate (72.1%)	“Sapna_2021” [38], “Baduci_2019” [26]	“Baduci_2019” [26]	17	0.2408 [0.2075; 0.2757]	low (45.9%)	+4.3%	−36.3%
type IV	19	0.1991 [0.1678; 0.2322]	moderate (52.7%)	“Baduci_2019” [26]	-	18	0.1916 [0.1631; 0.2217]	low (42.4%)	−3.8%	−19.5%
type V	19	0.0888 [0.0656; 0.1148]	moderate (56.8%)	“Weerapant_2010” [41]	“Weerapant_2010” [41]	18	0.0797 [0.0654; 0.0951]	minor (0.0%)	−10.3%	−100%
type VI	19	0.0879 [0.0596; 0.1204]	moderate (72.4%)	“Rana_2017” [2], “Weerapant_2010” [41]	-	17	0.0893 [0.0635; 0.1186]	moderate (59.3%)	+1.6%	−18.1%
bifurcation	19	0.9412 [0.8895; 0.9791]	high (88.0%)	“Davis_1958” [8], “Katz_1987” [9], “Baduci_2019” [26], “Agarwal_2022” [46]	-	15	0.9447 [0.9020; 0.9771]	moderate (73.0%)	+0.4%	−17.1%
trifurcation	12	0.1201 [0.0684; 0.1824]	moderate (72.7%)	“Rana_2017” [2]	-	11	0.1340 [0.0811; 0.1966]	moderate (65.0%)	+11.6%	−10.6%

## Data Availability

The data are available upon reasonable requests to the corresponding author (George Triantafyllou, georgerose406@gmail.com; Professor Maria Piagkou, piagkoumara@gmail.com).

## Supplementary Materials

The following supporting information can be downloaded at https://www.mdpi.com/article/10.3390/diagnostics14171862/s1, Figure S1: Influence analysis, baujat plot and leave-one-out analysis for FN Type I morphology [2,6,8,9,10,11,26,27,28,29,30,31,32,33,34,35,36,37,38,39,40,41,42,43,44,45,46,47]; Figure S2: Influence analysis, baujat plot and leave-one-out analysis for FN Type II morphology. Red dots showed the influential study [2,6,8,9,10,11,26,27,28,29,30,31,32,33,34,35,36,37,38,39,40,41,42,43,44,45,46,47]; Figure S3: Influence analysis, baujat plot and leave-one-out analysis for FN Type III morphology. Red dots showed the influential study [2,6,8,9,10,11,26,27,28,29,30,31,32,33,34,35,36,37,38,39,40,41,42,43,44,45,46,47]; Figure S4: Influence analysis, baujat plot and leave-one-out analysis for FN Type IV morphology [2,6,8,9,10,11,26,27,28,29,30,31,32,33,34,35,36,37,38,39,40,41,42,43,44,45,46,47]; Figure S5: Influence analysis, baujat plot and leave-one-out analysis for FN Type V morphology. Red dots showed the influential study [2,6,8,9,10,11,26,27,28,29,30,31,32,33,34,35,36,37,38,39,40,41,42,43,44,45,46,47]; Figure S6: Influence analysis, baujat plot and leave-one-out analysis for FN Type VI morphology [2,6,8,9,10,11,26,27,28,29,30,31,32,33,34,35,36,37,38,39,40,41,42,43,44,45,46,47]; Figure S7: Influence analysis, baujat plot and leave-one-out analysis for FN trunk bifurcation morphology [2,6,8,9,10,11,26,27,28,29,30,31,32,33,34,35,36,37,38,39,40,41,42,43,44,45,46,47]; Figure S8. Influence analysis, baujat plot and leave-one-out analysis for FN trunk trifurcation morphology [2,6,8,9,10,11,26,27,28,29,30,31,32,33,34,35,36,37,38,39,40,41,42,43,44,45,46,47].
