# Supplementary material for: Extratemporal Facial Nerve Interconnections and Trunk’s Variability: A Systematic Review with Meta-Analysis
Source: Diagnostics (Basel). 2024 Aug 26;14(17):1862. doi: 10.3390/diagnostics14171862 (PMC11393905; doi:10.3390/diagnostics14171862)

Figure S1. Influence analysis, baujat plot and leave-one-out analysis for FN Type I morphology [2,6,8–11,26–47].

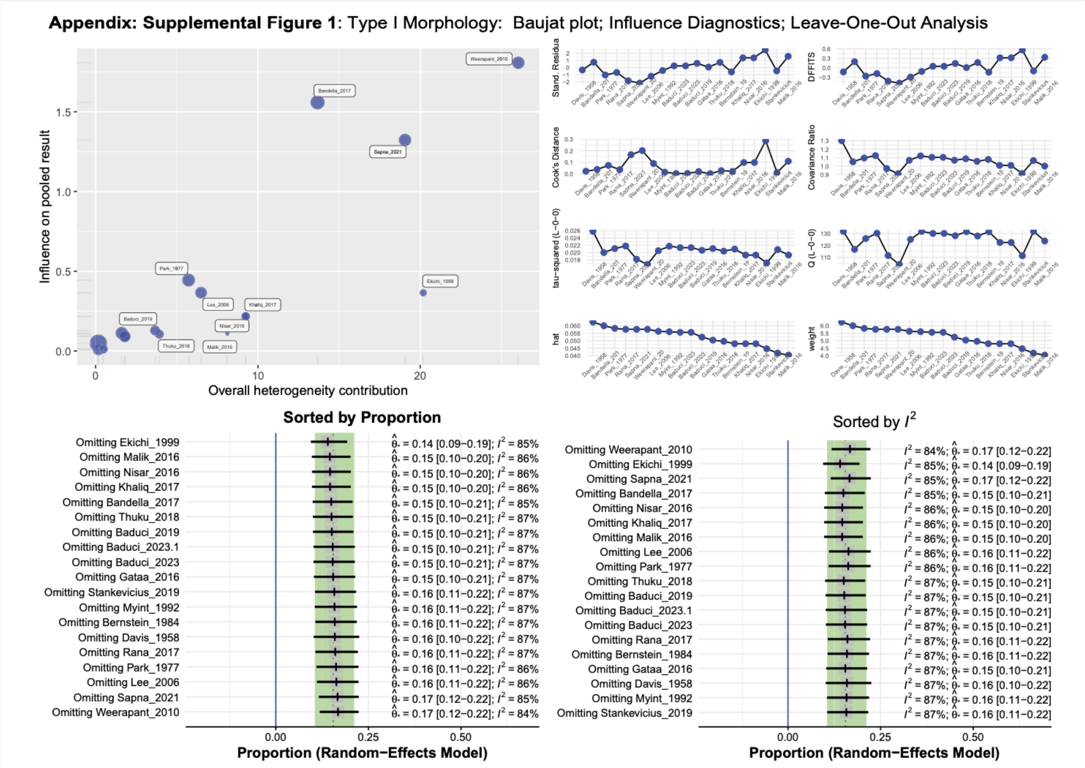

Figure S2. Influence analysis, baujat plot and leave-one-out analysis for FN Type II morphology. Red dots showed the influential study **[2,6,8–11,26–47]**.

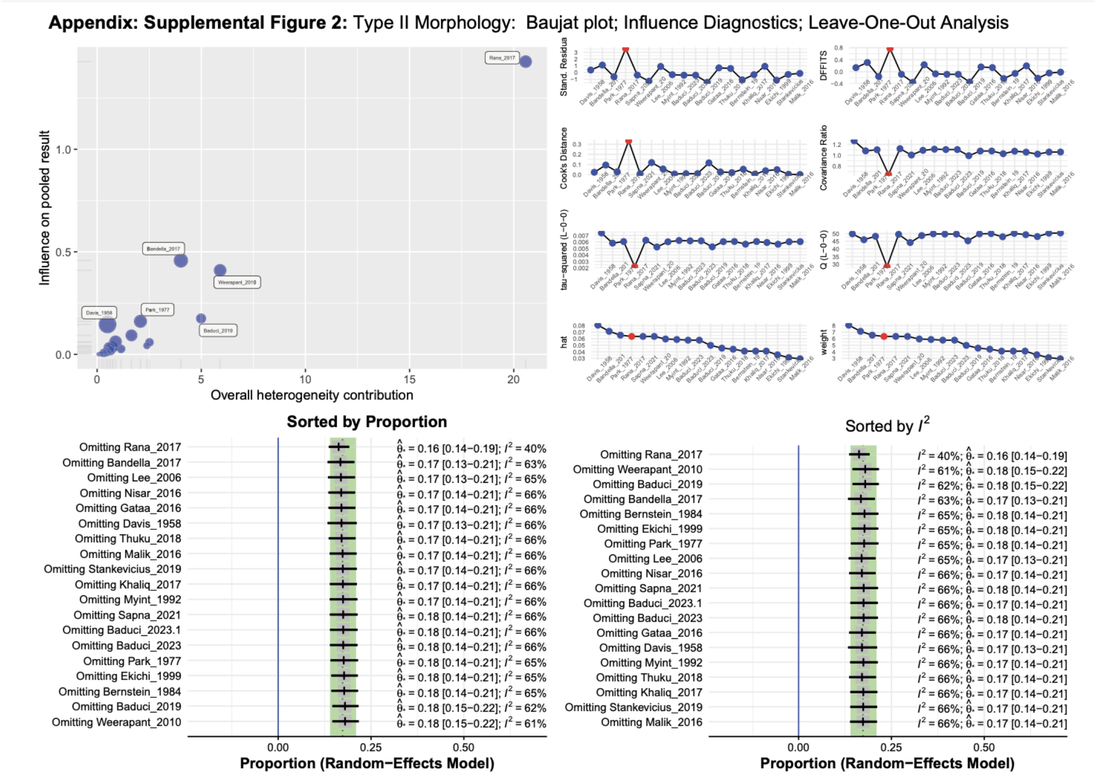

Figure S3. Influence analysis, baujat plot and leave-one-out analysis for FN Type III morphology. Red dots showed the influential study **[2,6,8–11,26–47]**.

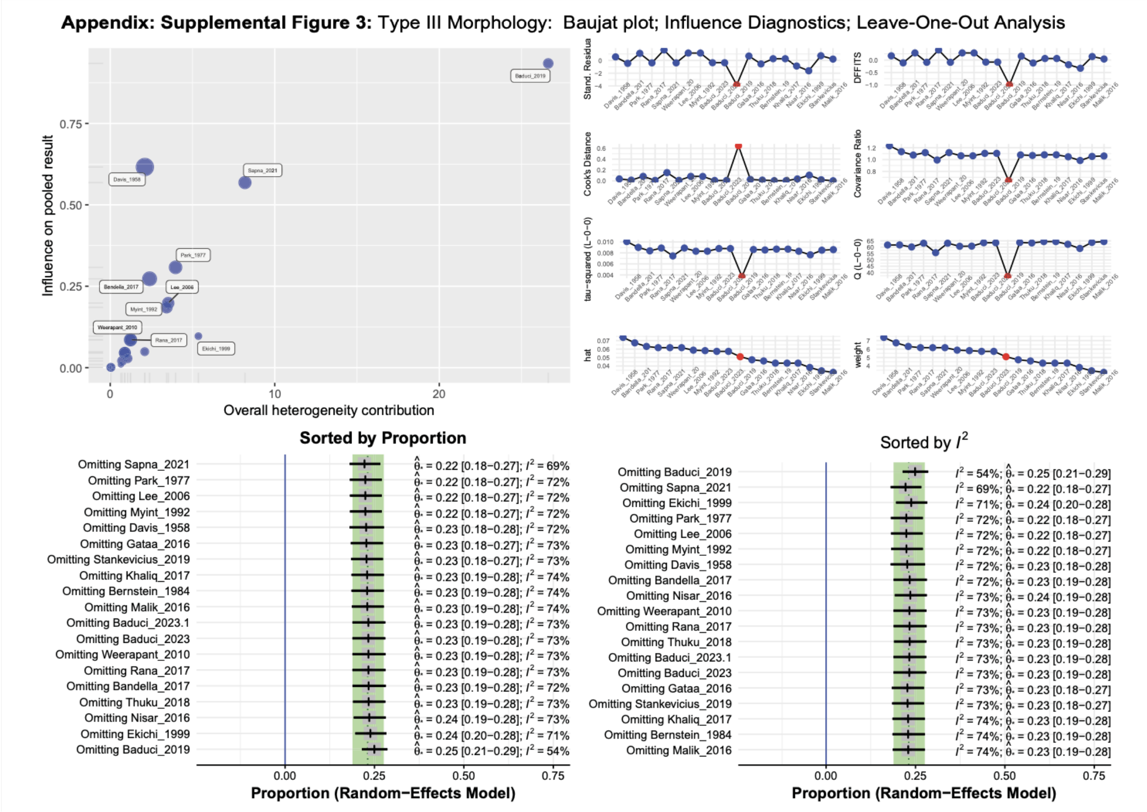

Figure S4. Influence analysis, baujat plot and leave-one-out analysis for FN Type IV morphology **[2,6,8–11,26–47]**.

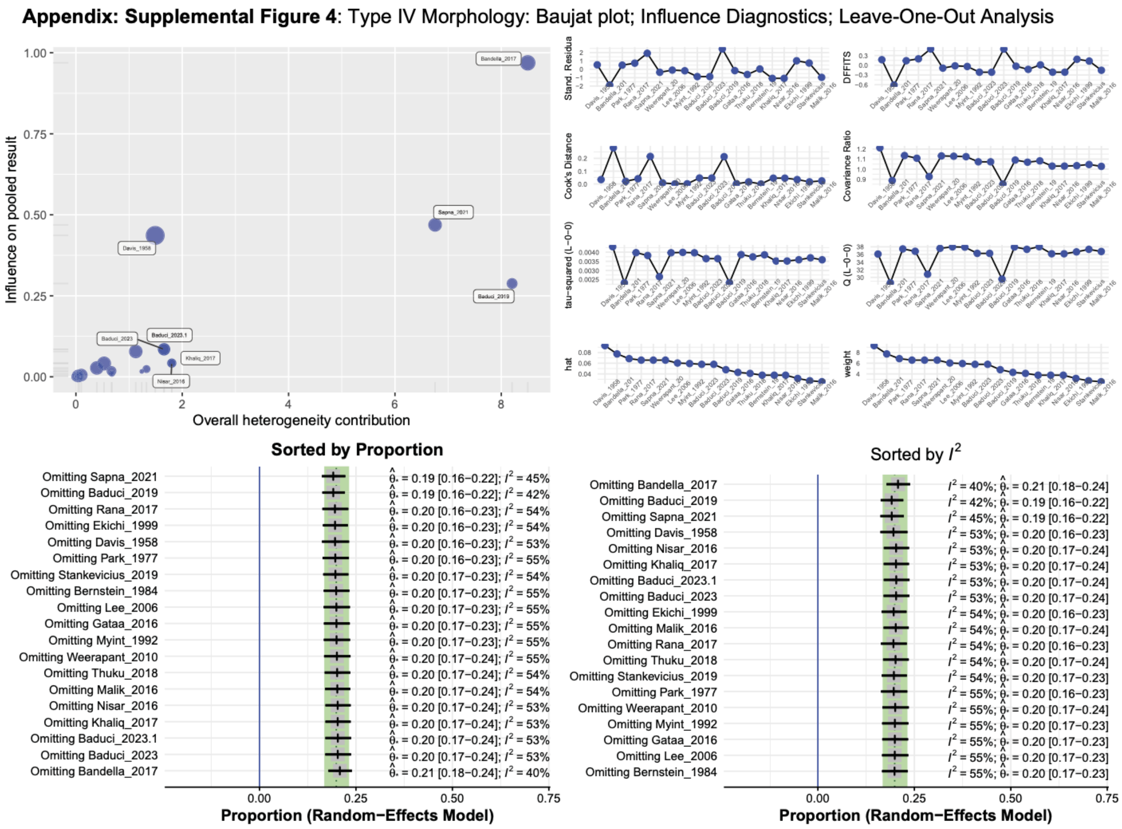

Figure S5. Influence analysis, baujat plot and leave-one-out analysis for FN Type V morphology. Red dots showed the influential study **[2,6,8–11,26–47]**.

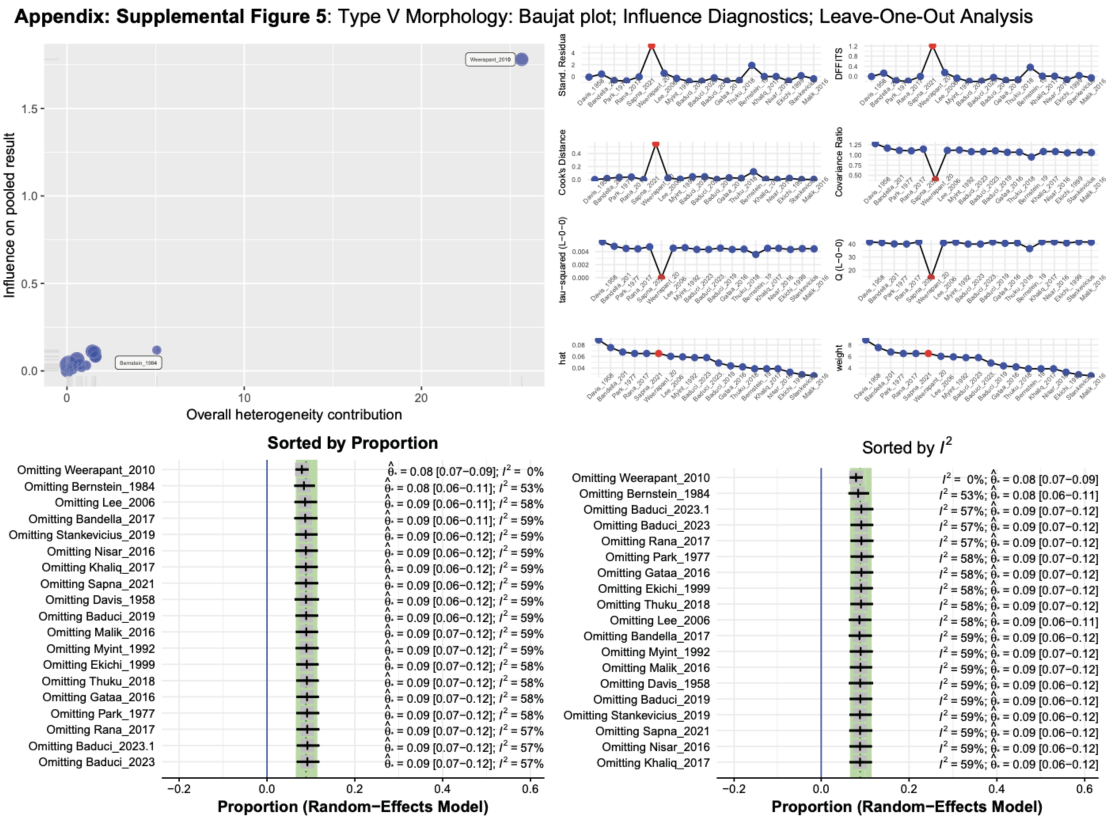

Figure S6. Influence analysis, baujat plot and leave-one-out analysis for FN Type VI morphology **[2,6,8–11,26–47]**.

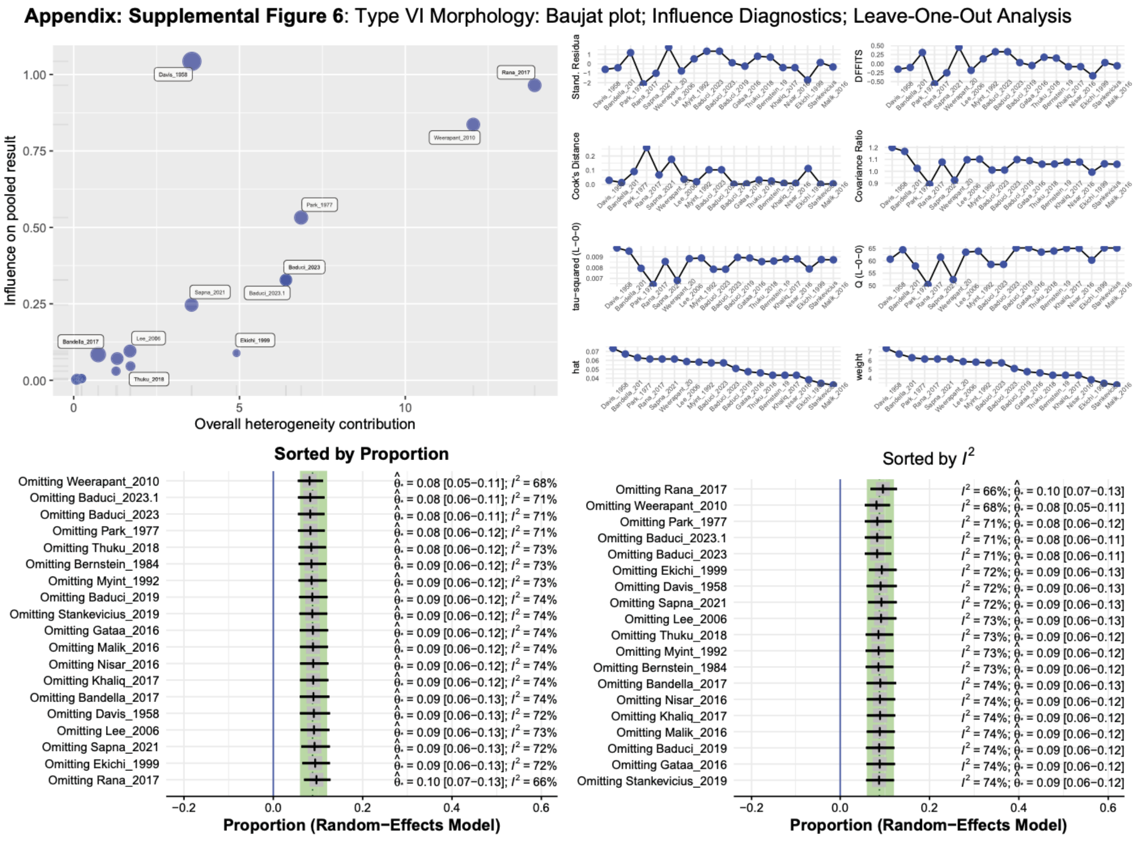

Figure S7. Influence analysis, baujat plot and leave-one-out analysis for FN trunk bifurcation morphology **[2,6,8–11,26–47]**.

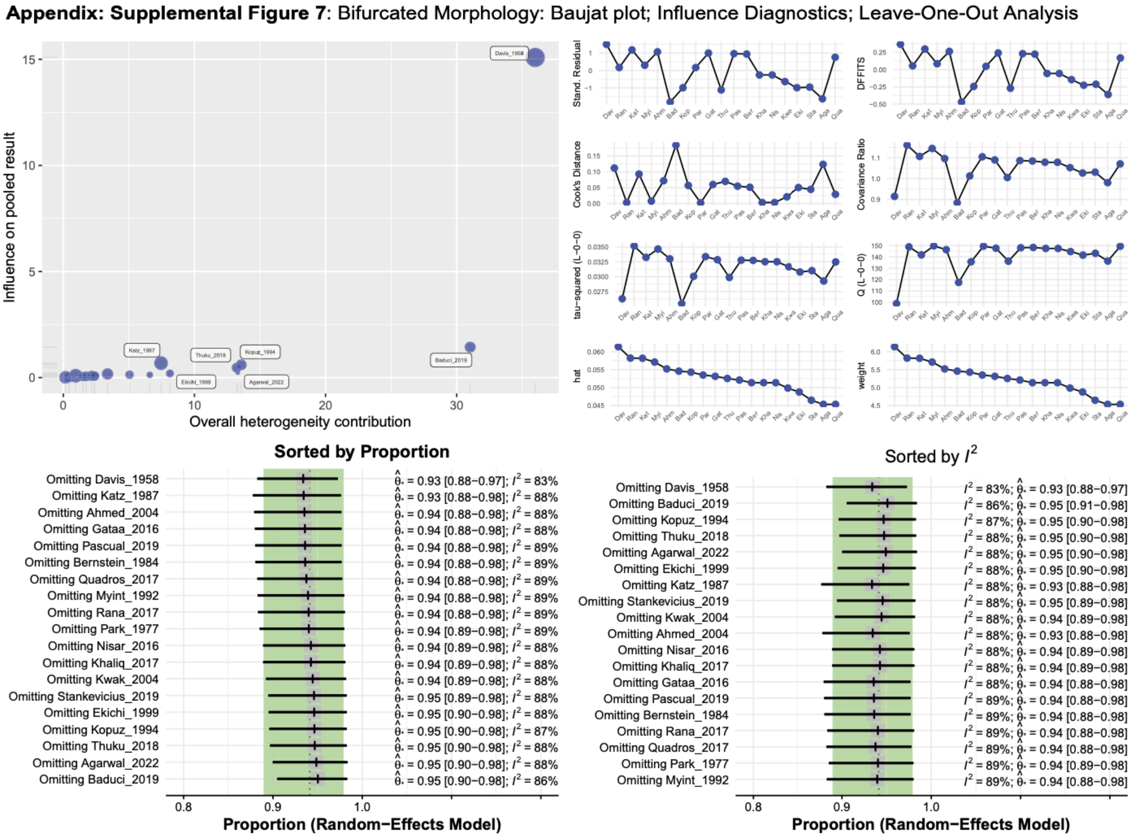

Figure S8. Influence analysis, baujat plot and leave-one-out analysis for FN trunk trifurcation morphology **[2,6,8–11,26–47]**.

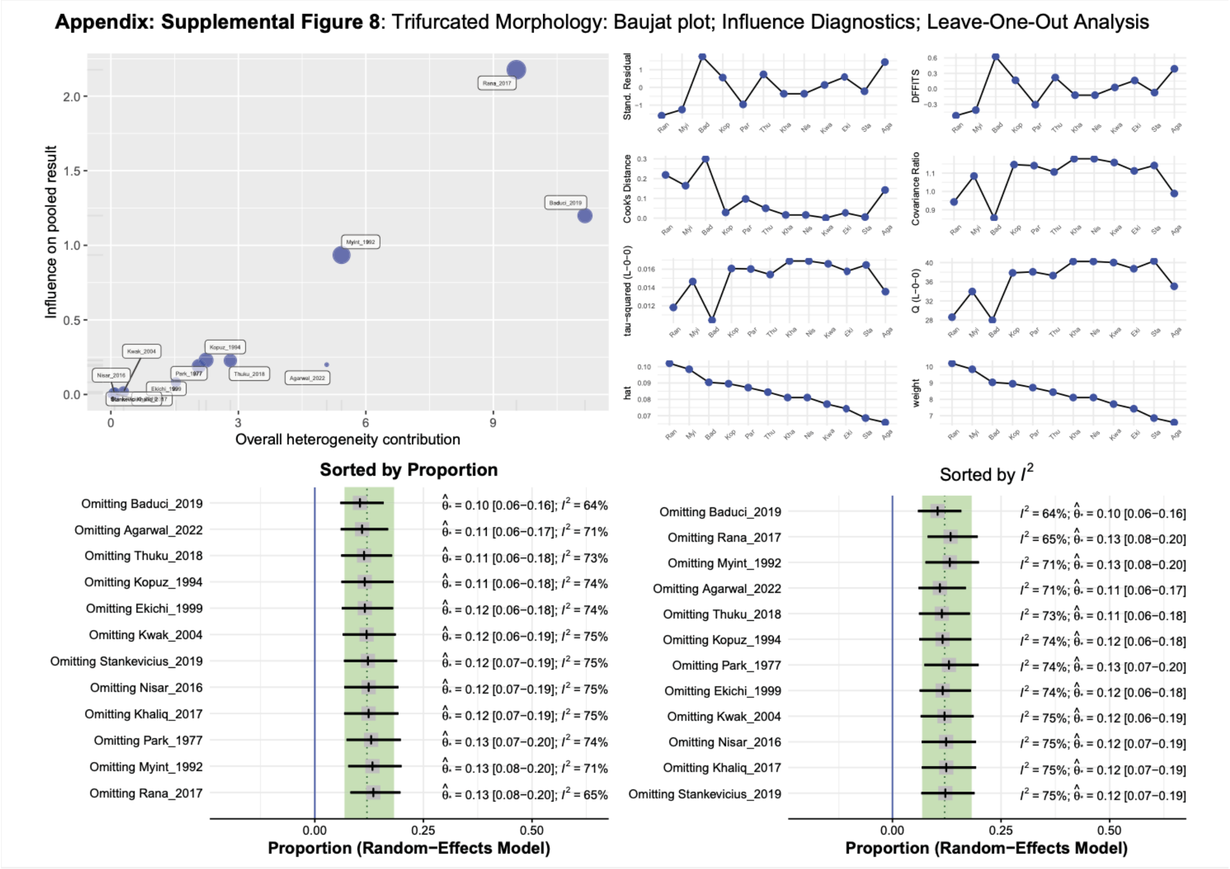

Supplement: Supplementary file 1 [file diagnostics-14-01862-s001.zip › diagnostics-3156073-supplementary.pdf]
